# Silent toxicity: A rare case of 5-fluorouracil-induced hyperammonemic encephalopathy

**DOI:** 10.18632/oncoscience.638

**Published:** 2025-12-23

**Authors:** Areti Kalfoutzou, Cleopatra Rapti, Eleftheria Bagiokou, Vasileios Kolintzikis, Vasileios Ramfidis

**Affiliations:** ^1^Second Propaedeutic Department of Internal Medicine, Attikon General Hospital, National and Kapodistrian University of Athens, Athens, Greece; ^2^Department of Medical Oncology, 251 Air Force General Hospital, Athens, Greece; ^3^Oncology Unit, 3rd Department of Internal Medicine, Athens General Hospital of Thoracic Diseases “Sotiria”, National and Kapodistrian University of Athens, Athens, Greece; ^4^Second Department of Medical Oncology, Agios Savvas Cancer Hospital, Athens, Greece

**Keywords:** hyperammonemia, encephalopathy, fluorouracil, neurotoxicity

## Abstract

Hyperammonemic encephalopathy (HE) is a rare but serious neurological condition characterized by an acute alteration in mental status due to elevated serum ammonia levels, occurring in the absence of known liver disease. The build-up of ammonia, a by-product of protein metabolism, in the bloodstream leads to its crossing of the blood-brain barrier, where it acts as a neurotoxin, causing potentially reversible brain damage. Chemotherapeutic agents such as 5-fluorouracil (5-FU) are known to cause drug-induced HE. Our case reports a 63-year-old woman who presented with several episodes of reduced consciousness shortly after 5-FU administration, highlighting the necessity of monitoring serum ammonia levels in patients treated with 5-FU who develop neurological symptoms, and the need for expert consultation in attempting a 5-FU rechallenge.

## INTRODUCTION

Hyperammonemic encephalopathy is characterized by a sudden alteration in mental status caused by elevated levels of ammonia, occurring in the absence of any known liver disease [1]. Most common causes of HE are metabolic disorders, particularly urea cycle disorders (UCD) and certain drugs. Hyperammonemic encephalopathy due to 5-FU is a rare but serious adverse event, with an incidence of 1% and a reported mortality rate of 17% [2, 3]. This case describes a middle-aged female with pancreatic adenocarcinoma treated with FOLFIRINOX, who experienced multiple episodes of mental status change shortly after 5-FU administration. Serum ammonia levels were found significantly elevated, while the liver function tests (LFT) and neuroimaging scans were unremarkable. The patient’s symptoms resolved after permanently stopping 5-FU and administering lactulose and intravenous fluids, therefore supporting the diagnosis of hyperammonemic encephalopathy due to 5-FU.

## CASE PRESENTATION

A 63-year-old Caucasian female was diagnosed with pancreatic adenocarcinoma in 2012. Her past medical history included epileptic seizures, diabetes mellitus and hypothyroidism. She underwent a Whipple pancreatectomy along with 6 cycles of adjuvant chemotherapy with gemcitabine.

Four years later, a follow-up MRI scan of the abdomen demonstrated a suspicious lesion measuring 25 mm in the pancreatic tail, strongly indicative of disease recurrence. Computed Tomography (CT) scans of the brain and chest were insignificant, and the patient was submitted to a total pancreatectomy and splenectomy, and “adjuvant-like” chemotherapy with FOLFIRINOX (5-Fluouracil, leucovorin, irinotecan and oxaliplatin). At this point, the patient’s medication list included the following: levetiracetam, lacosamide, pancrelipase, glucagon, long-acting insulin and insulin lispro.

Two days after the 4th cycle of chemotherapy, the patient was admitted due to an acute change in mental status. The patient’s daughter reported similar episodes of confusion and somnοlence in the patient, which typically occurred 3–4 days following each chemotherapy cycle and resolved spontaneously within a few days. On admission, the patient was afebrile, with no clinical signs of sepsis or dehydration. Neurological examination revealed a lethargic patient who barely responded to verbal commands (Glasgow Coma Scale – GCS: 11/15). No focal neurological deficits or signs of meningeal irritation were observed.

**Figure 1:**
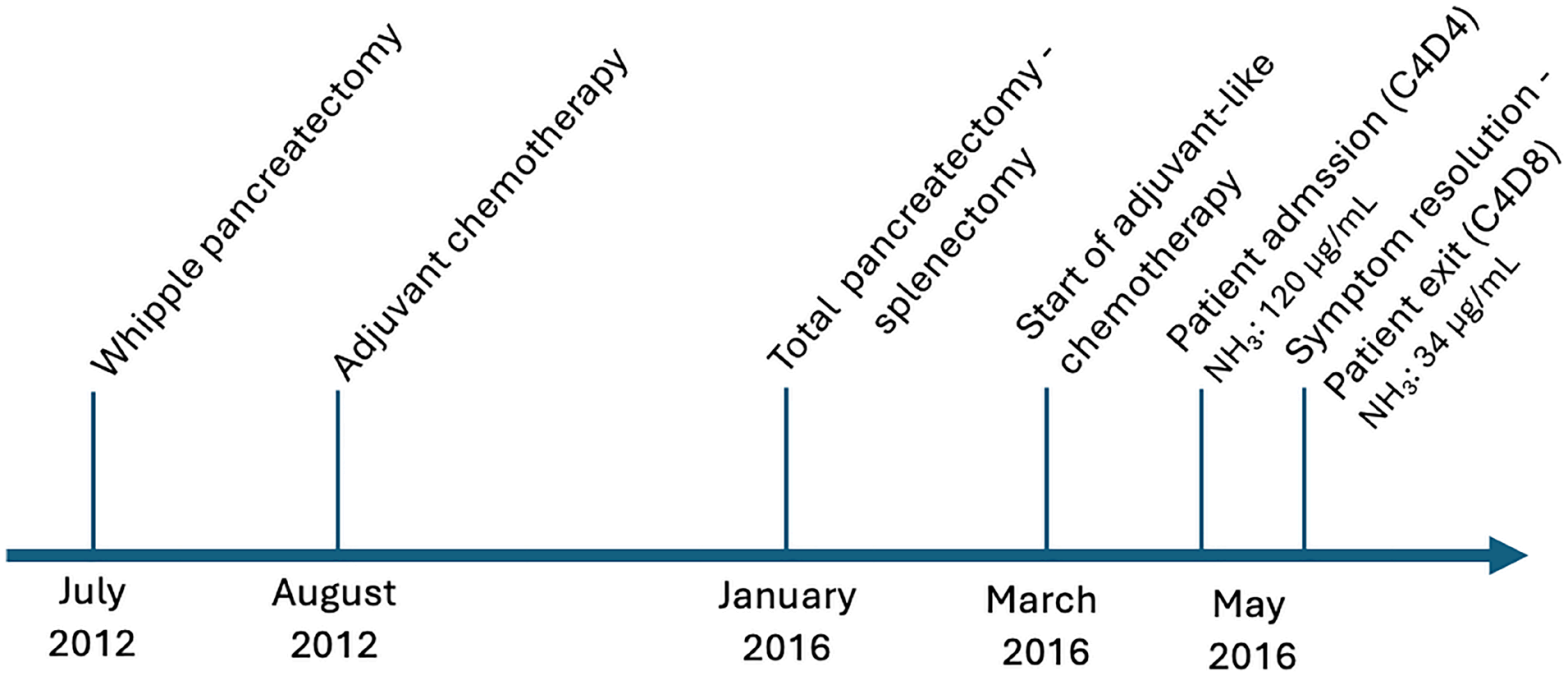
Timeline of key clinical events and management.

Laboratory examinations demonstrated normocytic normochromic anemia, along with normal liver function tests (LFT) (Table 1). Serum ammonia levels were markedly elevated (120 μg/mL, normal range: 0–32). Magnetic Resonance Imaging (MRI) scan of the brain and abdomen with intravenous contrast was unremarkable. An electroencephalogram (EEG) was negative for signs of epileptic activity. Lactulose was administered at a dose of 30mL per os daily, leading to a subsequent improvement in the patient’s mental status and serum ammonia levels (34 μg/mL) after 2 days. The Naranjo probability score was 7 (Table 2), classifying our case as a probable adverse drug reaction. The patient was discharged after 4 days in excellent clinical condition. Key clinical points are summarized in Figure 1.

**Table 1:** Laboratory examinations of the patient upon admission and discharge

Laboratory examination	Patient values (Day 1)	Patient values (Day 4)	Reference range
White Blood Cells	12.5	8.7	4–10 K/μL
Neutrophils	19.8	6.8	1.5–7 K/μL
Hemoblobin	10.2	9.4	12–16 g/dL
Hematocrit	34	31.2	36–46%
Platelets	435	374	140–440 K/μL
Blood Urea Nitrogen	28	34	15–54 mg/dL
Creatinine	0.6	0.7	0.55–1.2 mg/dL
Glucose	102	112	75–110 mg/dL
ALT	44	31	5–45 IU/L
AST	36	34	10–40 IU/L
γGT	59	55	10–60 IU/L
ALP	112	103	35–116 IU/L
Total bilirubin	1.09	1.07	0–1.3 mg/dL
Direct bilirubin	0.3	0.3	0–0.3 mg/dL
Albumin	3.5	3.3	3.5–5.5 g/dL
Na	138	135	137–150 mEq/L
K	4.1	3.7	3.5–5.3 mEq/L
Serum Ammonia	120	44	11–32 μg/mL

Abbreviations: ALT: Alanine Aminotransferase; AST: Aspartate Aminotransferase; γ-GT: Gamma-Glutamyl Transferase; ALP: Alkaline Phosphatase.

**Table 2:** Naranjo causality assessment for 5-fluorouracil (5-FU) [16]

Question	Score
1. Are there previous conclusive reports on this reaction?	Yes (+1)
2. Did the adverse event appear after the suspected drug was administered?	Yes (+2)
3. Did the adverse event improve when the drug was discontinued or a specific antagonist was administered?	Yes (+1)
4. Did the adverse event reappear when the drug was readministered?	Yes (+2)
5. Are there alternative causes that could on their own have caused the reaction?	Yes (−1)
6. Did the reaction reappear when a placebo was given?	Unknown (0)
7. Was the drug detected in blood or other fluids in concentrations known to be toxic?	Unknown (0)
8. Was the reaction more severe when the dose was increased or less severe when the dose was decreased?	Unknown (0)
9. Did the patient have a similar reaction to the same or similar drugs in any previous exposure?	Yes (+1)
10. Was the adverse event confirmed by any objective evidence?	Yes (+1)

A comprehensive review of the patient’s medication was conducted, and 5-Fluouracil (5-FU) was considered the primary cause of hyperammonemic encephalopathy, as the symptoms emerged shortly after the initiation of the 46-hour 5-FU infusion pump and resolved rapidly after its extraction. Notably, irinotecan, levetiracetam and lacosamide could have contributed to the patient’s symptoms since they are associated with hyperammonemia. Applying the Naranjo algorithm yielded scores of 3 (possible) for irinotecan, 1 (possible) for levetiracetam, and 1 (possible) for lacosamide, thereby identifying 5-FU as the most likely cause of HE. Permanent discontinuation of 5-FU and irinotecan was decided, and the patient will be closely monitored for hyperammonia related to levetiracetam or lacosamide. A detailed molecular analysis is currently being processed to identify actionable gene alterations for potential treatment options.

## DISCUSSION

Hyperammonemic encephalopathy (HE) is defined as the sudden onset of neurological manifestations due to the elevation of serum ammonia, in the absence of known hepatotoxicity [1]. Excess ammonia in the bloodstream can cross the blood-brain barrier and act as a neurotoxin, leading to rapid onset of neurological symptoms by disrupting neurotransmission and causing astrocyte edema [3]. Physiologically, ammonia is detoxified in the liver via the urea cycle, while astrocytes convert ammonia to glutamine via glutamine synthetase [4]. Metabolic disorders, particularly urea cycle disorders, and certain drugs are among the most common causes of excess ammonia in the body [5].

Drug-induced hyperammonemic encephalopathy (HE) has been linked to several medications, including anticonvulsants like valproic acid, levetiracetam, and lacosamide, as well as chemotherapeutic agents including 5-fluorouracil, capecitabine, gemcitabine, irinotecan, and tyrosine kinase inhibitors such as sunitinib, imatinib, sorafenib, and regorafenib [1, 2, 6, 7]. 5-fluouracil, in particular, yields catabolites (fluoro-β-alanine, monofluoroacetate) that depress tricarboxylic acid (TCA) cycle flux, reduce adenosine triphosphate (ATP), and secondarily impair the urea cycle, predisposing to hyperammonemia [8]. A study published by Balcerac in 2022 demonstrated that, among 2924 reported cases of drug-induced hyperammonemia from 1967 to 2020, 5-FU was the second most common agent, accounting for 301 cases globally [2].

Additionally, it has been hypothesised that chemotherapy-induced diarrhea caused has a protective effect against the risk of hyperammonemic encephalopathy by promoting to the rapid excretion of ammonia [9]. Furthermore, gene alterations that affect the metabolism of 5-FU, such as dihydropyrimidine dehydrogenase (DPD) deficiency and TYMS gene polymorphisms, are suspected to play a role in HE-pathogenesis caused by 5-FU [4]. Pre-existing liver disease, dehydration, sepsis, cachexia, renal failure, chronic constipation and several drug-drug interactions are known patient-related risk factors for 5-FU induced HE [9–11].

Clinical presentation of 5-FU induced HE includes a wide range of symptoms such as loss of appetite, nausea/vomiting, confusion, seizures, or psychiatric disorders [12]. Subtle manifestations including agitation, loss of concentration, or urinary incontinence may occur [13]. The symptoms usually emerge 2 days after the drug initiation and resolve within 2–10 days, whereas few patients experience long-term neurologic sequelae [12].

Agents known to reduce ammonia reabsorption, such as lactulose, antibiotics that lower the ammonia-producing bacteria in the intestinal flora, particularly rifaximin, along with dietary protein restriction and intravenous fluids, are indicated for the treatment of hyperammonemic encephalopathy [5, 6, 14]. Intravascular volume expansion improves renal perfusion, GFR, urine flow, and distal sodium delivery, thereby enhancing ammonium trapping and excretion [15]. Branched amino acids may also be indicated, while hemodialysis is reserved for more severe cases [9]. Additionally, uridine triacetate has been proposed as an antidote to severe 5-FU toxicity; however, it has not been explored as a treatment option for HE [12]. Recent literature, including the 2023 case report and review by Kurniawan et al., similarly emphasizes prompt recognition, discontinuation of 5-FU, supportive care, and careful consideration of rechallenge in selected cases, but only with close collaboration with an expert in metabolic diseases [12].

In our case, the clinical presentation in the absence of known liver disease or acute hepatotoxicity, as indicated by the normal laboratory and imaging tests, as well as the elevated serum ammonia levels, raised the suspicion of drug-induced hyperammonemic encephalopathy. The patient’s symptoms emerged soon after the initiation and resolved rapidly after discontinuing the 5-FU infusion. The recurrence of symptoms with each chemotherapy cycle, along with the lack of an alternative explanation, further supported our presumed diagnosis. Interestingly, in a study by Boilève et al. including 30 patients with 5-FU induced HE, serum ammonia levels were measured in 50% of patients, despite the lack of an alternate differential diagnosis [12]. This highlights the need for routinely measuring serum ammonia in any patient treated with 5-FU presenting with acute neurological manifestations [12].

## CONCLUSIONS

Hyperammonemia caused by chemotherapeutic or targeted agents, while rare, is a recognized adverse event in cancer patients and should be considered in any patient with neurological symptoms following 5-FU administration. Clinicians should be particularly aware of this potentially fatal adverse event, remain vigilant when administering chemotherapeutic agents such as 5-FU and capecitabine, and be prepared to discontinue therapy if clinically indicated.
